# Supplementary material for: Circulating nucleosomes as epigenetic biomarkers in pancreatic cancer
Source: Clin Epigenetics. 2015 Oct 7;7:106. doi: 10.1186/s13148-015-0139-4 (PMC4597435; doi:10.1186/s13148-015-0139-4)
Supplement: Additional file 1: — Performance of individual nucleosome assays. ROC curves for each nucleosome assay in cancer vs. healthy or benign and cancer vs. healthy groups. [file 13148_2015_139_MOESM1_ESM.pdf]

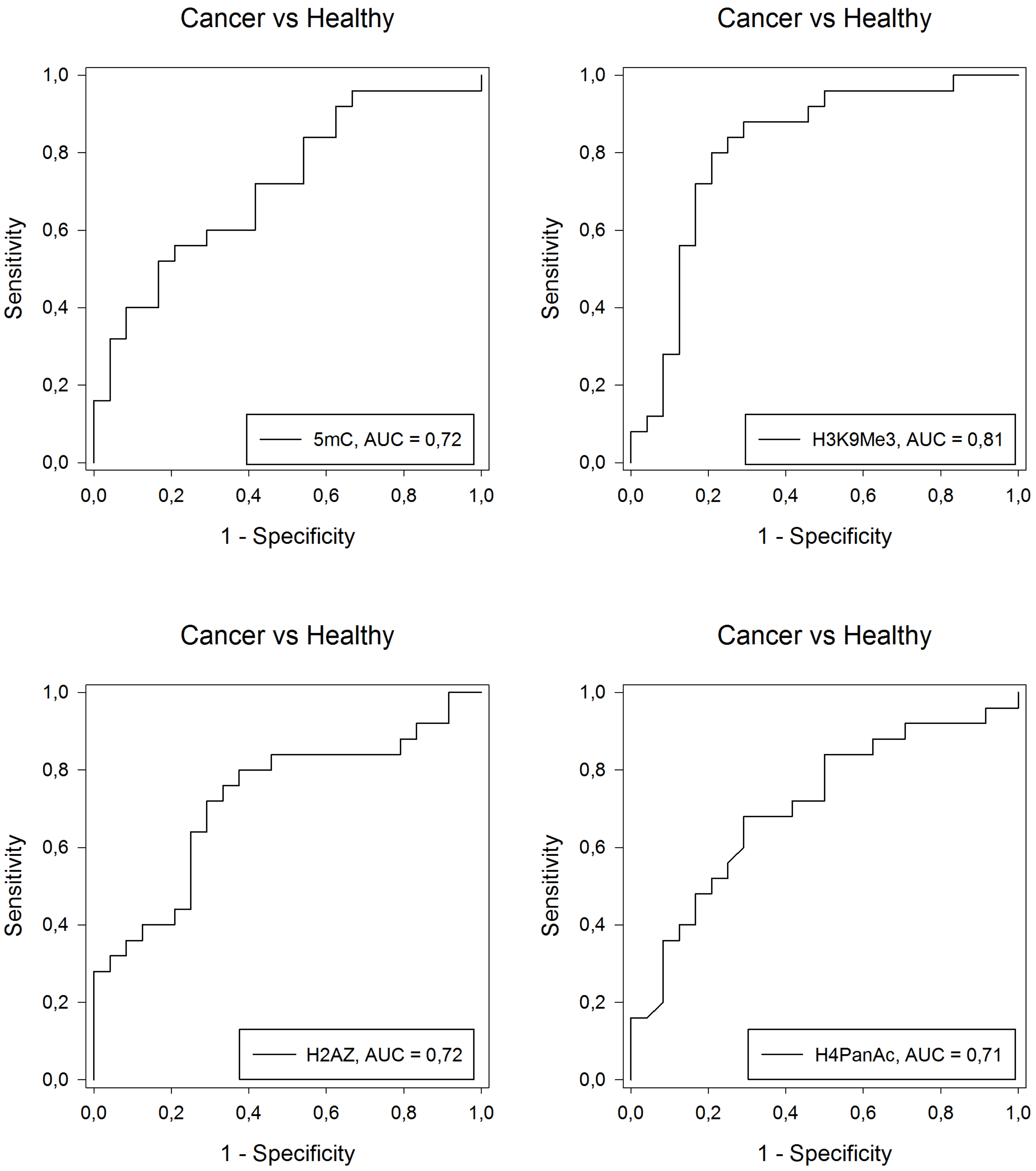
Cancer vs Healthy
Sensitivity
1 - Specificity
5mC, AUC = 0,72
Cancer vs Healthy
Sensitivity
1 - Specificity
H3K9Me3, AUC = 0,81
Cancer vs Healthy
Sensitivity
1 - Specificity
H2AZ, AUC = 0,72
Cancer vs Healthy
Sensitivity
1 - Specificity
H4PanAc, AUC = 0,71

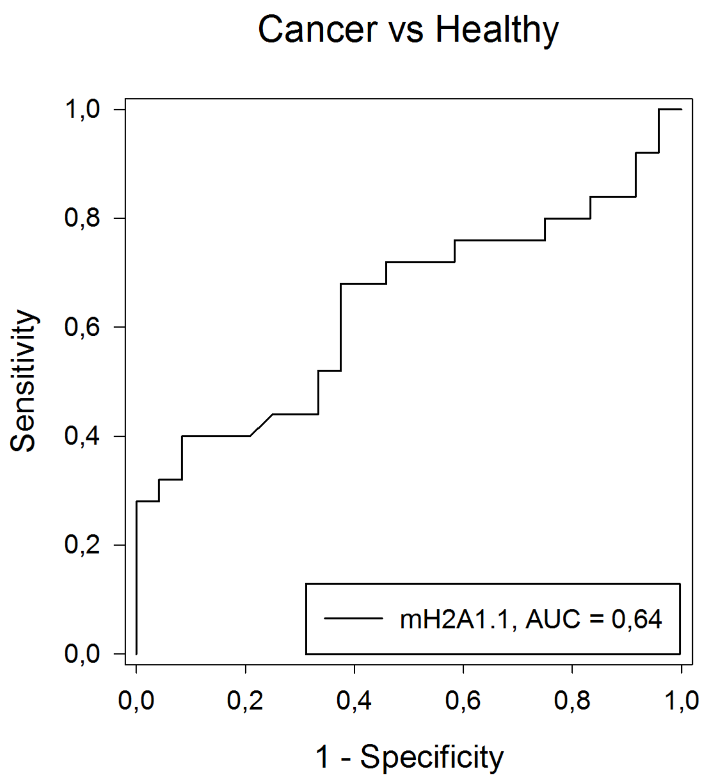

Cancer vs Healthy
Sensitivity
1 - Specificity
1,0
0,8
0,6
0,4
0,2
0,0
0,0
0,2
0,4
0,6
0,8
1,0
mH2A1.1, AUC = 0,64

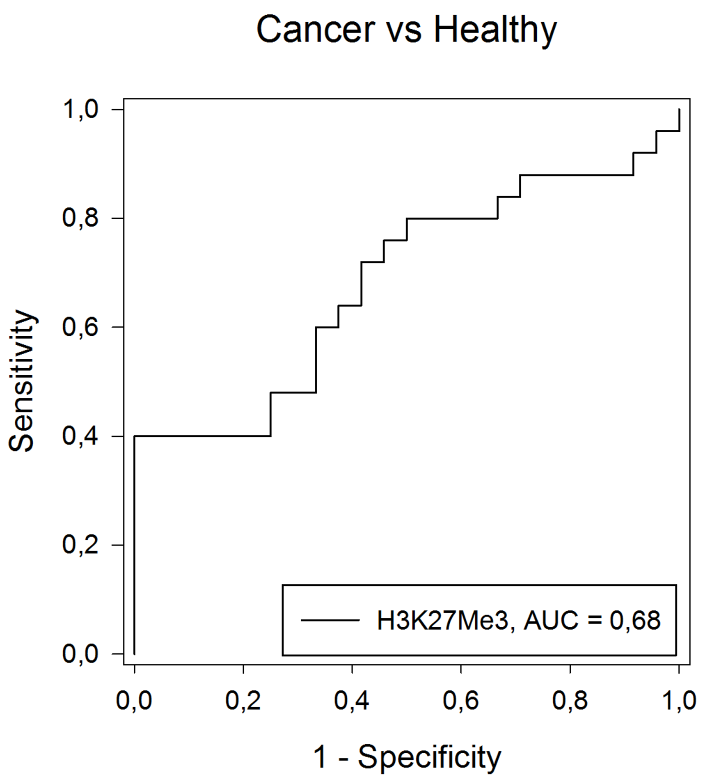

Cancer vs Healthy
Sensitivity
1 - Specificity
1,0
0,8
0,6
0,4
0,2
0,0
0,0
0,2
0,4
0,6
0,8
1,0
H3K27Me3, AUC = 0,68

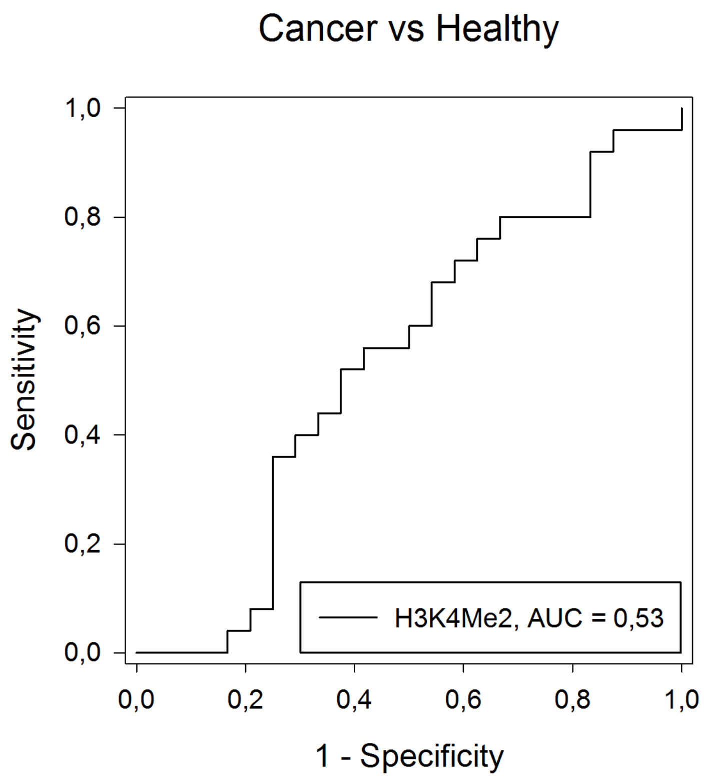

Cancer vs Healthy
Sensitivity
1 - Specificity
1,0
0,8
0,6
0,4
0,2
0,0
0,0
0,2
0,4
0,6
0,8
1,0
H3K4Me2, AUC = 0,53

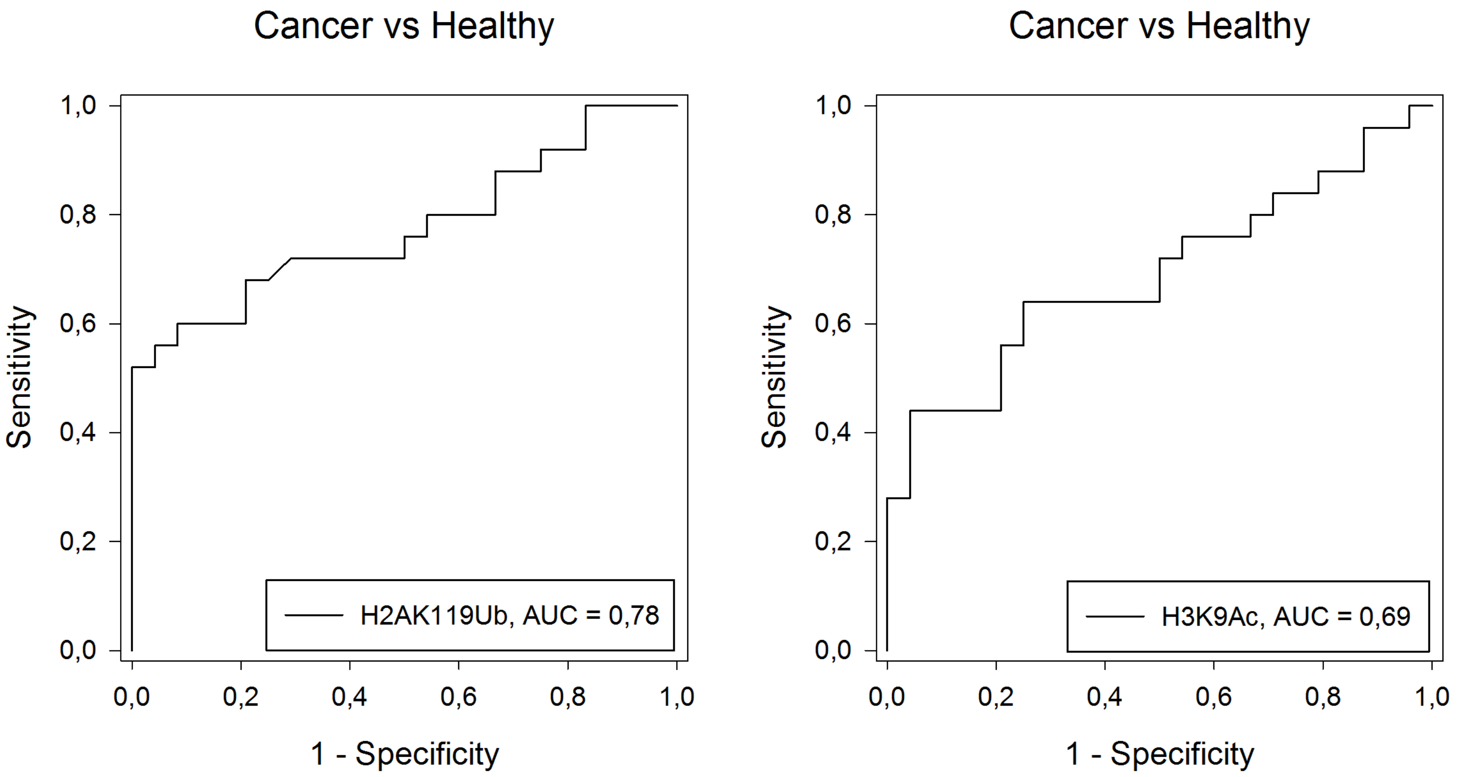
Cancer vs Healthy
Sensitivity
1,0
0,8
0,6
0,4
0,2
0,0
0,0
0,2
0,4
0,6
0,8
1,0
1 - Specificity
H2AK119Ub, AUC = 0,78
Cancer vs Healthy
Sensitivity
1 - Specificity
H3K9Ac, AUC = 0,69

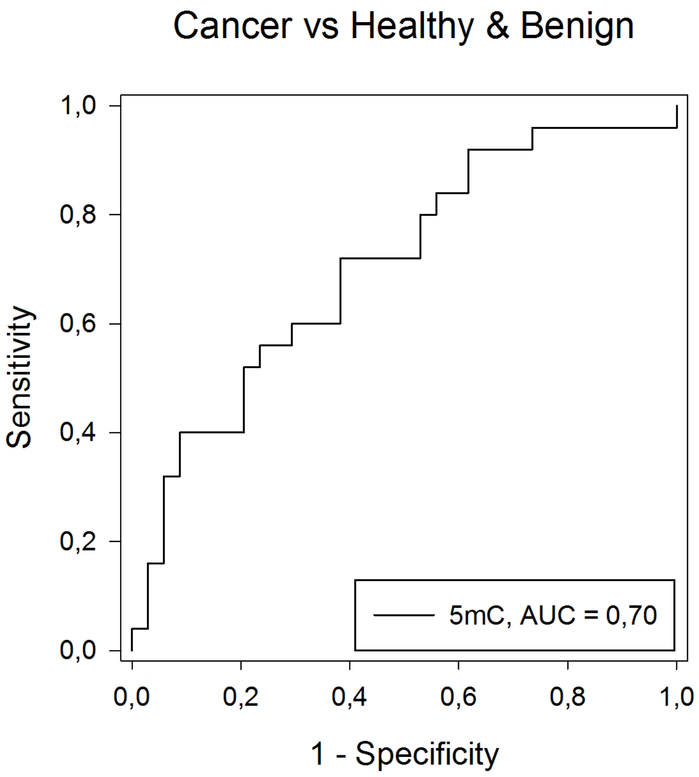
Cancer vs Healthy & Benign
Sensitivity
1 - Specificity
5mC, AUC = 0,70
0,0
0,2
0,4
0,6
0,8
1,0

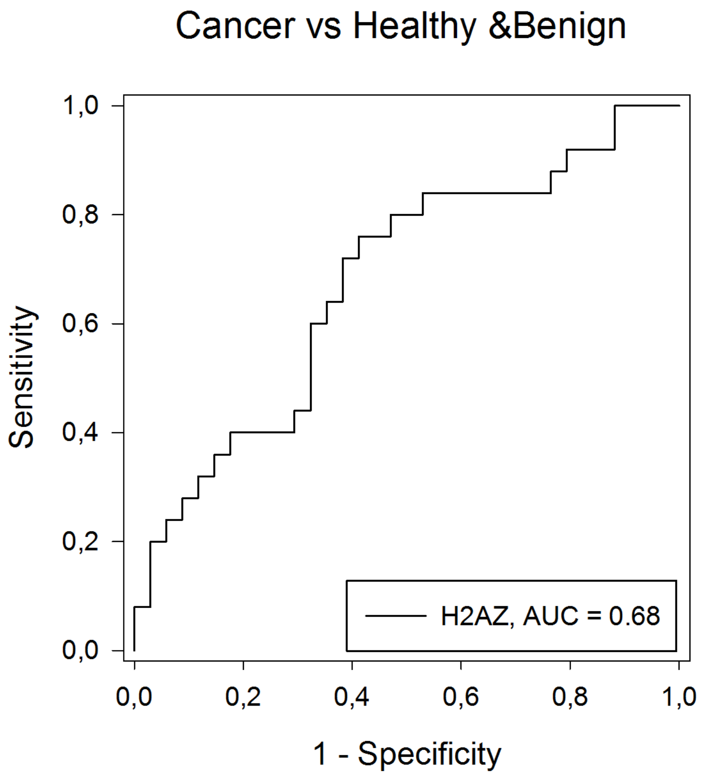
Cancer vs Healthy &Benign
Sensitivity
1 - Specificity
H2AZ, AUC = 0.68
0,0
0,2
0,4
0,6
0,8
1,0

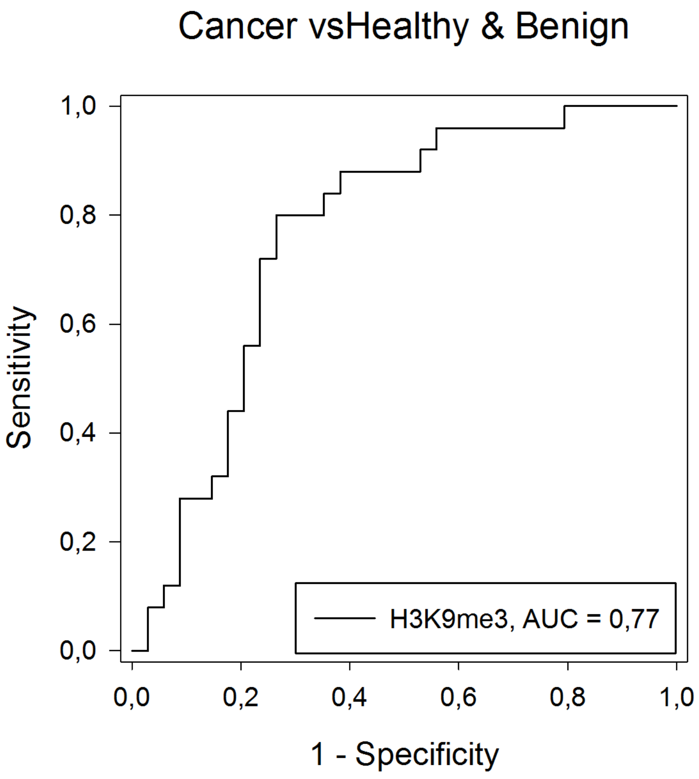
Cancer vsHealthy & Benign
Sensitivity
1 - Specificity
H3K9me3, AUC = 0,77
0,0
0,2
0,4
0,6
0,8
1,0

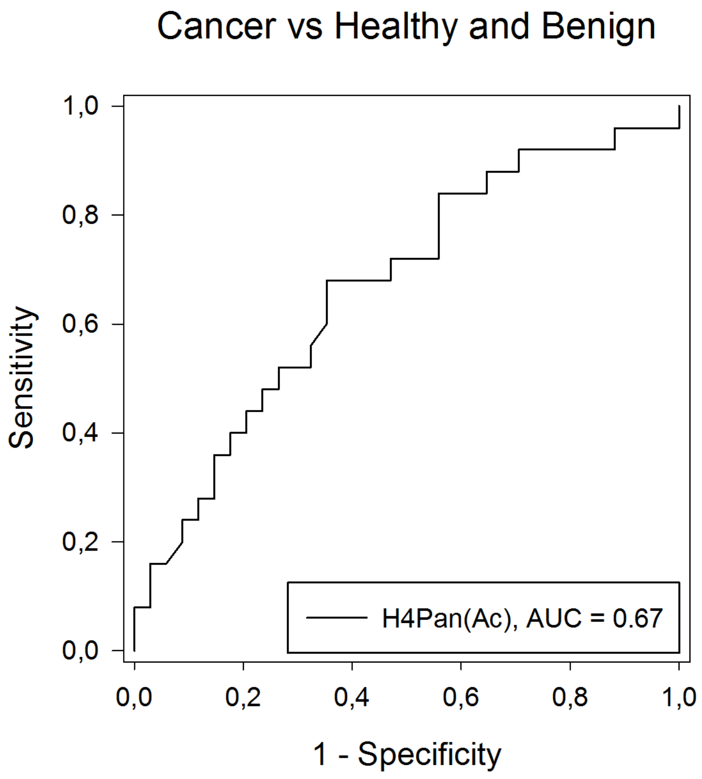
Cancer vs Healthy and Benign
Sensitivity
1 - Specificity
H4Pan(Ac), AUC = 0.67
0,0
0,2
0,4
0,6
0,8
1,0

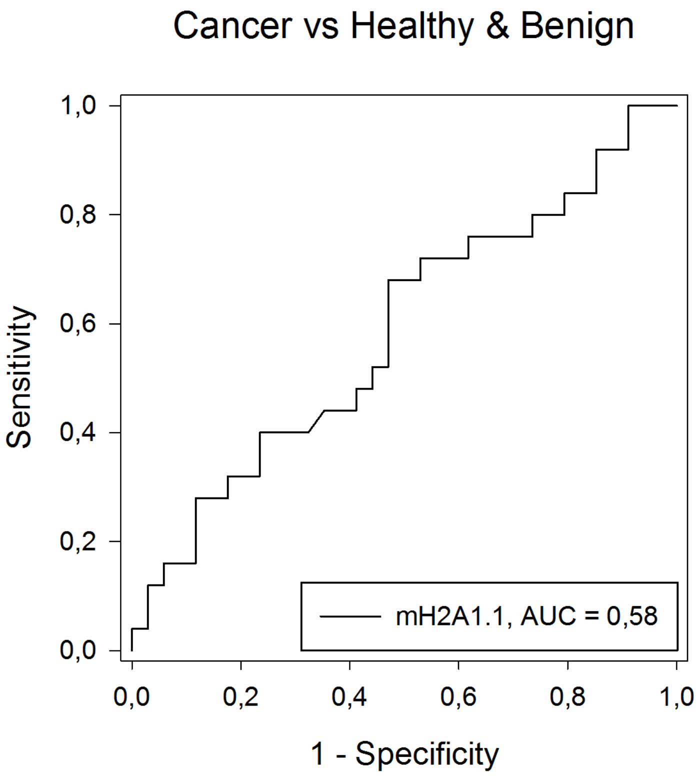
Cancer vs Healthy & Benign
Sensitivity
1 - Specificity
mH2A1.1, AUC = 0,58
0,0
0,2
0,4
0,6
0,8
1,0

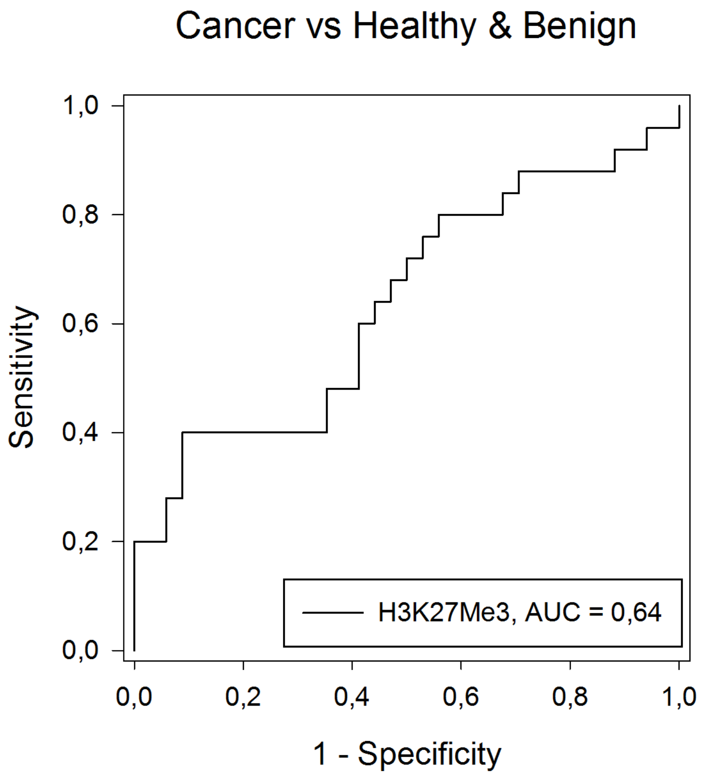
Cancer vs Healthy & Benign
Sensitivity
1 - Specificity
H3K27Me3, AUC = 0,64
0,0
0,2
0,4
0,6
0,8
1,0

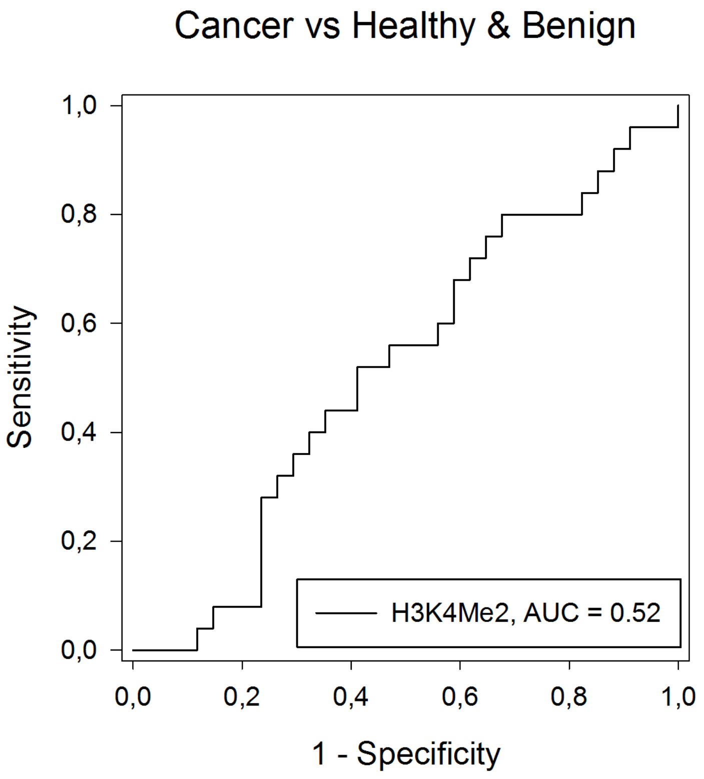
Cancer vs Healthy & Benign
Sensitivity
1 - Specificity
H3K4Me2, AUC = 0.52
0,0
0,2
0,4
0,6
0,8
1,0

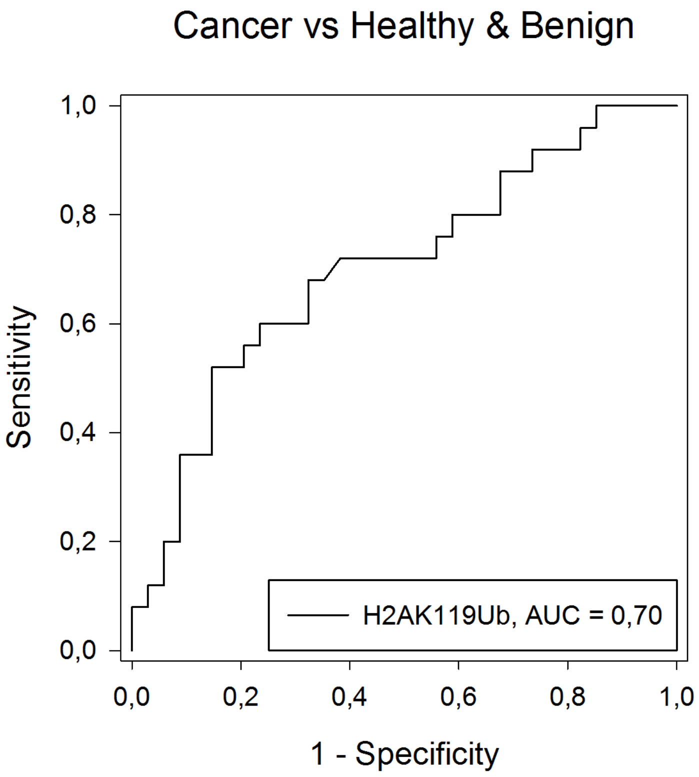
Cancer vs Healthy & Benign
Sensitivity
1 - Specificity
0,0
0,2
0,4
0,6
0,8
1,0
H2AK119Ub, AUC = 0,70

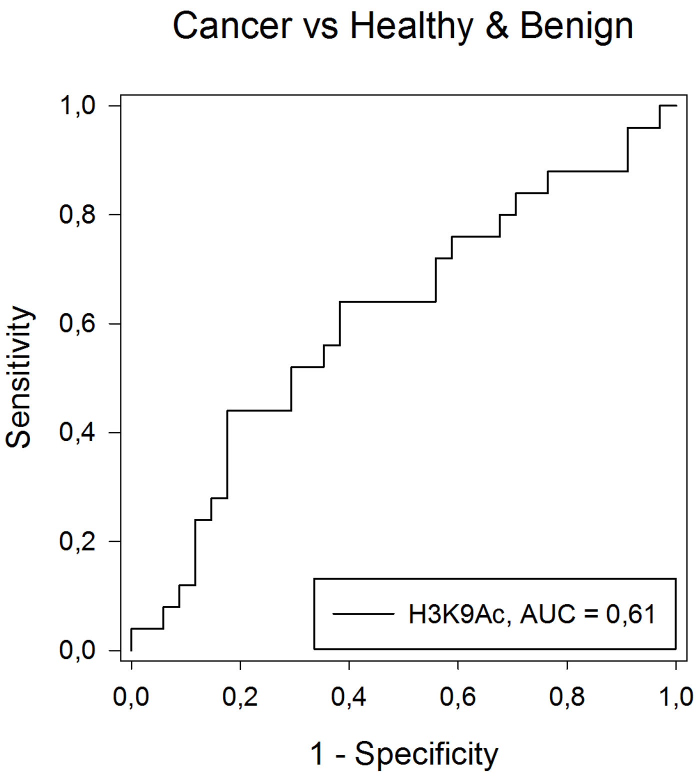
Cancer vs Healthy & Benign
Sensitivity
1 - Specificity
0,0
0,2
0,4
0,6
0,8
1,0
H3K9Ac, AUC = 0,61
